# Role of the Notch ligand DLL4 in the immune response of children with *Mycoplasma pneumoniae* pneumonia

**DOI:** 10.1590/S1678-9946202567065

**Published:** 2025-10-03

**Authors:** Heting Dong, Zhiao Du, Yaru Liao, Jiying Sun, Huiming Sun, Peng Mo, Ge Dai, Li Huang, Feng Huang, Chuangli Hao, Zhengrong Chen, Yongdong Yan

**Affiliations:** 1 Soochow University, Children’s Hospital, Department of Respiratory Medicine, Suzhou, China; 2 Shandong University, Children’s Hospital, Department of Respiratory Medicine, Jinan, China

**Keywords:** Mycoplasma pneumoniae, Pneumonia, Notch, DLL4, Cytokines, Lymphocyte subsets

## Abstract

Mycoplasma pneumoniae pneumonia (MPP) is a common pediatric respiratory infection linked to excessive immune-inflammatory responses. This study investigated the role of the Notch ligand DLL4 in the immunopathogenesis of MPP by assessing its expression in peripheral blood mononuclear cells of affected children. A total of 128 children with MPP and 35 controls were recruited. PBMCs were analyzed for the expression of Notch ligands (Jagged1, Jagged2, DLL1, DLL4) using real-time PCR. Lymphocyte subsets were assessed via flow cytometry, and cytokine levels were measured using ELISA. Clinical data were compared between severe and mild MPP cases, and correlations between DLL4 expression and immune indicators were evaluated. DLL4 expression was significantly higher in the MPP and severe MPP groups than in controls (P < 0.01). MPP patients showed lower CD3+ and CD3+CD4+ lymphocyte levels, and higher CD3+CD8+ and CD3-CD19+ levels compared with controls (P < 0.001). Plasma levels of IFN-γ, IL-17, and IL-36α were elevated in MPP patients (P < 0.001), whereas IL-4 and IL-10 levels were reduced (P < 0.01). Severe cases had higher IFN-γ, IL-17, and IL-36α levels than mild cases (P < 0.05). DLL4 expression positively correlated with plasma IFN-γ and IL-17 levels in MPP patients (P < 0.05). Elevated DLL4 expression in MPP patients, particularly in severe cases, suggests its role in enhancing Th1/Th17-mediated immune responses while suppressing Th2 pathways. Such findings implicate the Notch signaling pathway, via DLL4, in the immunopathogenesis of MPP and highlight its potential as a therapeutic target for modulating immune responses in severe MPP.

## INTRODUCTION

*Mycoplasma pneumoniae* (MP) is a leading cause of community-acquired pneumonia in children^1^. The incidence of *M. pneumoniae* pneumonia (MPP) is rising, with a younger age of onset^2^. MPP is characterized by extensive immune-mediated pathology, including excessive inflammation, lymphocyte subset dysregulation, and cytokine storms, which often lead to severe complications, such as bronchiolitis obliterans and extrapulmonary manifestations^3,4^. The lack of a cell wall in MP makes treatment difficult. There is intrinsic resistance to beta-lactam antibiotics, and macrolides are the only first-line treatment option^5^. However, rising macrolide resistance (up to 90% in Asia) and the lack of targeted immunomodulatory strategies underscore the urgent need to elucidate immune mechanisms in MPP pathogenesis^6,7^.

The Notch signaling pathway, which is a conserved regulator of cell differentiation and immune responses, has emerged as a critical player in infectious and inflammatory diseases^8^. In particular, the Notch ligand DLL4 has been implicated in polarizing T-cell responses toward pro-inflammatory T helper 1 (Th1) and Th17 phenotypes, while suppressing Th2-mediated anti-inflammatory pathways^9^. This balance is pivotal in MPP, in which excessive Th1/Th17 responses correlate with tissue damage and disease severity^10^. DLL4 overexpression in dendritic cells enhances IFN-γ and IL-17 production, mirroring the cytokine profiles observed in severe MPP^11^. Despite these advances, the role of DLL4 in pediatric MPP remains unexplored, and its potential as a therapeutic target has not been investigated in infectious contexts.

Given the current global challenge of macrolide-resistant MP strains^12-14^, identifying new therapeutic targets is crucial. Investigating DLL4 expression and its association with immune indicators may offer a novel avenue for immune-targeted treatments in MPP, particularly for severe cases in which excessive Th1/Th17 responses predominate. Therefore, this study aims to evaluate the role of DLL4 in the immunopathogenesis of MPP by examining its expression in peripheral blood mononuclear cells and correlating it with immune cell subsets, cytokine profiles, and disease severity. Our findings may provide clinically relevant insights into the potential of DLL4 as a biomarker or therapeutic target for immune modulation in pediatric MPP.

## MATERIALS AND METHODS

### Ethics

This study was approved by the Ethics Committee of the Children’s Hospital of Soochow University (process Nº 2023CS231) and was conducted in accordance with the principles outlined in the Declaration of Helsinki.

### Study design and study population

A total of 128 children diagnosed with Mycoplasma pneumoniae pneumonia (MPP) and treated at the Department of Respiratory Medicine at the Children’s Hospital Affiliated to Soochow University, from March 2015 to July 2016, were enrolled. Peripheral blood and nasopharyngeal aspirates were collected within 24 h of admission. As a control group, 35 children undergoing elective surgeries, for conditions such as inguinal hernia or polydactyly, in the Department of Surgery at the same hospital during the same period were included. The controls had no personal or family history of allergies or other allergic diseases, no infectious diseases in the preceding four weeks, and no prior use of medication. Written informed consent for blood testing was obtained from the parents of all participants. The children with MPP were categorized into severe and mild MPP groups based on their clinical condition, and the severe MPP group also fulfilled the diagnostic criteria for severe pneumonia^15^.

### Evaluation criteria for MP infection

The criteria for diagnosing MP infection were based on both serological and molecular evidence. Diagnosis was confirmed if either of the following conditions was met: (1) if a single serum MP-IgM level was ≥ 1.1 S/CO, or a fourfold increase or decrease in MP-IgG and/or MP-IgM levels between two serum samples collected seven to 14 days apart; or (2) if the MP DNA copy number in nasopharyngeal aspirates or bronchoalveolar lavage fluid was ≥ 1.0 × 10^5^ copies/mL.

### Exclusion criteria for MPP

The exclusion criteria were as follows: (1) a positive viral antigen or nucleic acid test in respiratory secretions; (2) clinical manifestations or additional evidence indicating typical bacterial pneumonia; (3) a positive purified protein derivative test or the presence of *Mycobacterium tuberculosis* in a sputum smear; (4) high-risk factors or clinical and imaging findings that are consistent with fungal pneumonia; (5) a history of recurrent respiratory tract infections; (6) other systemic diseases, such as congenital heart disease, connective tissue disease, immunodeficiency disorders, or diabetes; (7) continuous use of systemic glucocorticoids for ≥ 1 week within the past three months; (8) congenital bronchopulmonary dysplasia; and (9) participation in experimental drug or device trials.

### Classification criteria for MPP severity

MPP severity was classified as either mild or severe based on the established criteria for the severity classification of community-acquired pneumonia. This study did not include critically ill children with MPP.

### Detection of MP

#### Procedure for DNA Extraction and PCR Detection

Nasopharyngeal aspirates or bronchoalveolar lavage fluid were collected and centrifuged at 12,000 rpm for 5 min. The supernatant was discarded, and the sediment at the bottom of the tube was retained, labeled, and stored at −80 °C for subsequent DNA extraction. The stored samples were thawed at room temperature and mixed with 50 µL of DNA extraction solution, which was thoroughly mixed and centrifuged again at 12,000 rpm for 5 min. Two microliters of the extracted DNA were then added to a polymerase chain reaction (PCR) tube as the template for the reaction. The sample was centrifuged at 7,500 rpm for 30 s before being transferred to a real-time fluorescence quantitative PCR (RT-PCR) instrument for analysis. The RT-PCR cycling conditions were as follows: 95 °C for 5 min, followed by 40 cycles of 95 °C for 10 s, 55 °C for 30 s, and 72 °C for 40 s, with fluorescence collected at 55 °C during each cycle. The RT-PCR instrument automatically analyzed the results, which were expressed in Ct values. The criteria for interpreting the results were as follows: (I) a Ct value less than 38 indicated a positive result; (II) a Ct value between 38 and 39 required re-testing, and if the result remained within this range, it was considered negative; and (III) a Ct value greater than 39 was classified as negative.

## Detection of MP-specific antibodies (MP-IgG and MP-IgM) by enzyme-linked immunosorbent assay (ELISA)

The detection of MP-specific antibodies (MP-IgG and MP-IgM) was performed using ELISA. A fully quantitative method was employed for MP-IgG detection, while a semiquantitative method was used for MP-IgM detection. The absorbance values corresponding to the antibody concentrations of three standard sera were used to construct a standard curve for MP-IgG detection. The serum concentration of MP-IgG was then calculated based on this curve, which had the following result interpretation: (i) positive, IgG ≥ 22 RU/mL; (ii) suspected, 16 RU/mL ≤ IgG ≤ 22 RU/mL; and (iii) negative, IgG < 16 RU/mL. For suspected results, a second sample was collected for retesting before discharge (at least seven days apart) to observe changes in the antibody titer. The semiquantitative method for MP-IgM detection involved calculating the ratio of the absorbance values of the control serum or children’s samples to that of the standard product. The result interpretation for MP-IgM was as follows: (i) positive, ratio ≥ 1.1 S/CO; (ii) suspected, 0.8 S/CO ≤ ratio < 1.1 S/CO; and (iii) negative, ratio < 0.8 S/CO. Similarly, a second sample was collected for retesting suspected results before discharge (at least seven days apart) to assess changes in the antibody titer.

## Detection of Notch ligand expression levels in peripheral blood mononuclear cells (PBMCs)

PBMCs were isolated from blood samples. Total RNA was extracted, followed by reverse transcription to synthesize complementary DNA (cDNA). The resulting cDNA products were analyzed using RT-PCR. The primer sequences used for the detection of Notch ligands and the internal reference gene (GAPDH) were as follows:

hJAG1: sense, 5'-CAACGGCGAGTCCTTTAC-3'; antisense, 5'-CGGTAGCCATTGATCTCAT-3'

hJAG2: sense, 5'-AGCTGGAACGAGACGAGTGT-3'; antisense, 5'-TCTTGCCACCAAAGTCATCA-3'

hDLL1: sense, 5'-GGTCATCCTTGTCCTCATGCT-3'; antisense, 5'-CCGCCTTCTTGTTGGTGTTC-3'

hDLL4: sense, 5'-TGAGGTGCGGACATCCATC-3'; antisense, 5'-CTGCCCACAAAGCCATAAGG-3'

hGAPDH: sense, 5'-AGAAGGCTGGGGCTCATTTG-3'; antisense, 5'-AGGGGCCATCCACAGTCTTC-3'

The RT-PCR results were determined based on the melting curve and expressed as cycle threshold (Ct) values. GAPDH was used as the internal reference gene to normalize the data. The expression levels of Notch signaling pathway ligands were calculated using the 2-∆CT method, in which the ligand expression level was defined as 2-∆CT.

## Detection of the proportions of peripheral blood lymphocyte subsets and cytokines

The proportions of lymphocyte subsets in peripheral blood, including CD3+, CD3+CD4+, CD3+CD8+, and CD3-CD19+ cells, were determined using flow cytometry. Additionally, the expression levels of cytokines, including interferon-gamma (IFN-γ), interleukin-10 (IL-10), IL-17, IL-4, and IL-36α, were measured using ELISA.

## Statistical analysis

Statistical analyses were performed using the SPSS statistical software package (version 17.0, IBM, Armonk, NY, USA). Initial data evaluation included tests for normal distribution and homogeneity of variance. Quantitative data following a normal distribution were expressed as mean ± standard deviation (SD). For comparisons between two groups, a t-test was used for data with homogeneous variance, while a t’-test was applied for data with nonhomogeneous variance. Non-normally distributed quantitative data were shown as medians, and comparisons between two groups were performed using the Wilcoxon rank-sum test. The data were expressed as rates, and group comparisons were conducted using the χ^2^ test. Pearson linear correlation analysis was utilized to explore relationships between two variables. For correlation analysis involving DLL4, the values were log-transformed (lg[DLL4]) before analysis. A P-value of < 0.05 was statistically significant.

## RESULTS

### Comparison of the clinical characteristics of children with MPP

A total of 128 children with MPP with a mean age of 4.14 ± 3.19 years were included in the study. Among them, 52 children (40.6%) had severe MPP and 76 children (59.4%) had mild MPP. The fever duration was 6.72 ± 5.89 days in the severe MPP group and only 2.58 ± 2.94 days in the mild MPP group. Moreover, the hospitalization time was 8.56 ± 2.87 days in the severe MPP group and 7.34 ± 1.87 days in the mild MPP group (*P* = 0.000 and 0.027). The severe MPP group had significantly higher median values of peripheral white blood cells (WBC), neutrophil percentage, and C-reactive protein (CRP) compared with the mild MPP group (all *P* < 0.01). Regarding the humoral immune indexes, the severe MPP group had significantly higher levels of peripheral blood immunoglobulins (IgG and IgM) compared with the mild MPP group (both *P* < 0.05). However, no significant difference in IgA was observed between the two groups. About 78.8% of the children in the severe MPP group had lobar pneumonia, whereas only 22.4% in the mild MPP group had lobar pneumonia. Moreover, the children in the severe MPP group were more likely to have pleural effusion than those in the mild MPP group (Supplementary Table S1).

### Comparison of Notch ligand expression levels in PBMCs

**Figure 1:**
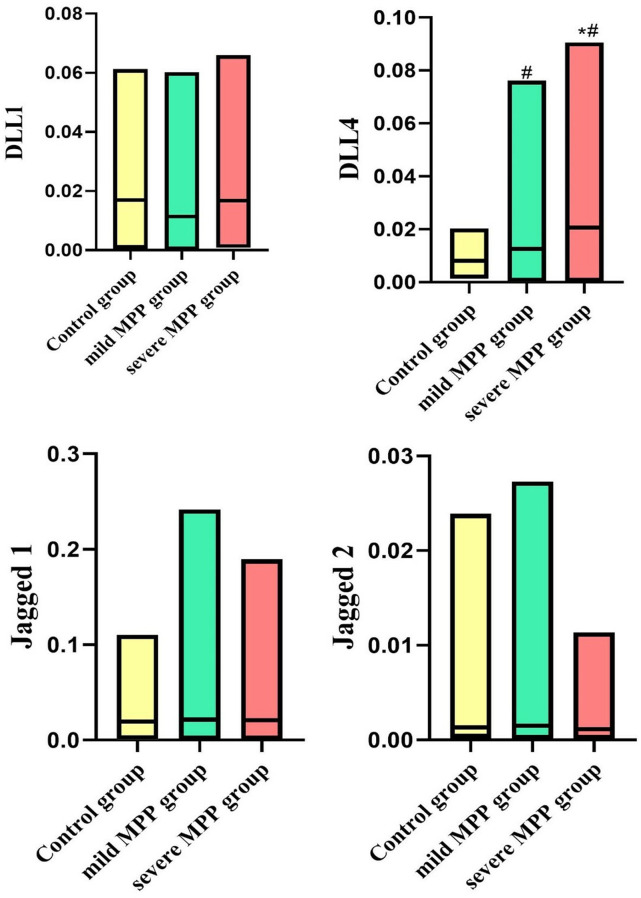
Comparison of Notch ligand expression levels in PBMCs. #*P* < 0.05 compared with the control group; *P < 0.05 compared with the mild MPP group.

The mRNA expression levels of the Notch ligand DLL4 in the PBMCs of the MPP group were generally higher, in which the severe MPP group had higher mRNA expression levels of the Notch ligand DLL4 compared with the mild MPP group (*P* = 0.01). However, no statistically significant differences in the expression levels of Jagged1, Jagged2, and DDL1 were observed among groups (all *P* > 0.05) (Figure 1).

### Comparison of the proportions of peripheral blood lymphocyte subsets

**Figure 2:**
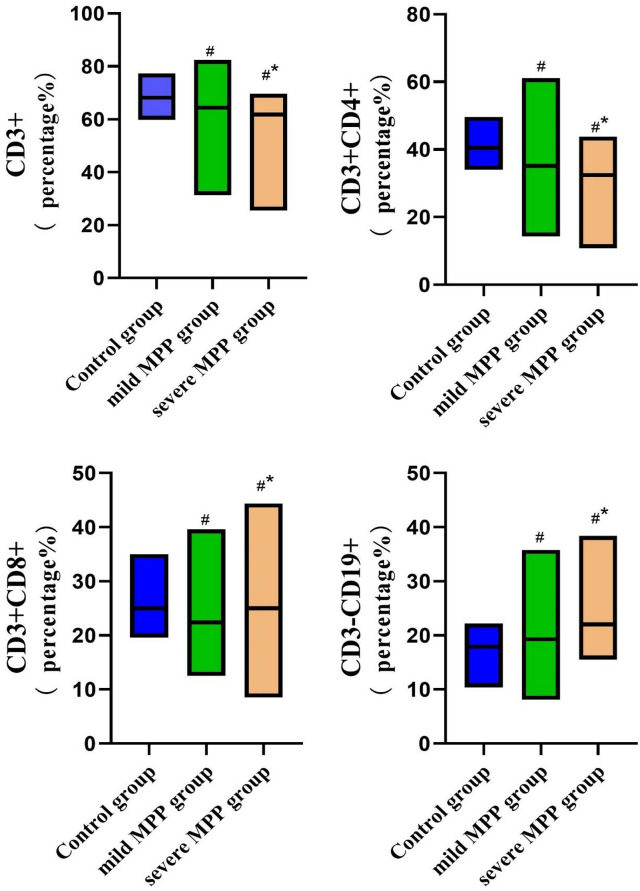
Comparison of the proportions of peripheral blood lymphocyte subpopulations. #*P* < 0.05 compared with the control group; *P < 0.05 compared with the mild MPP group.

The MPP group had lower levels of peripheral blood lymphocyte subsets CD3+ and CD3+CD4+ cells and higher levels of CD3+CD8+ and CD3-CD19+ cells compared with the control group (all *P* < 0.05). The severe MPP group had lower levels of peripheral blood lymphocyte subsets CD3+ and CD3+CD4+ cells (*P* < 0.05) and higher levels of CD3+CD8+, and CD3-CD19+ cells compared with the mild MPP group (*P* < 0.05) (Figure 2).

### Comparison of the expression levels of plasma IFN-γ, IL-4, IL-10, IL-17, and IL-36α

**Figure 3:**
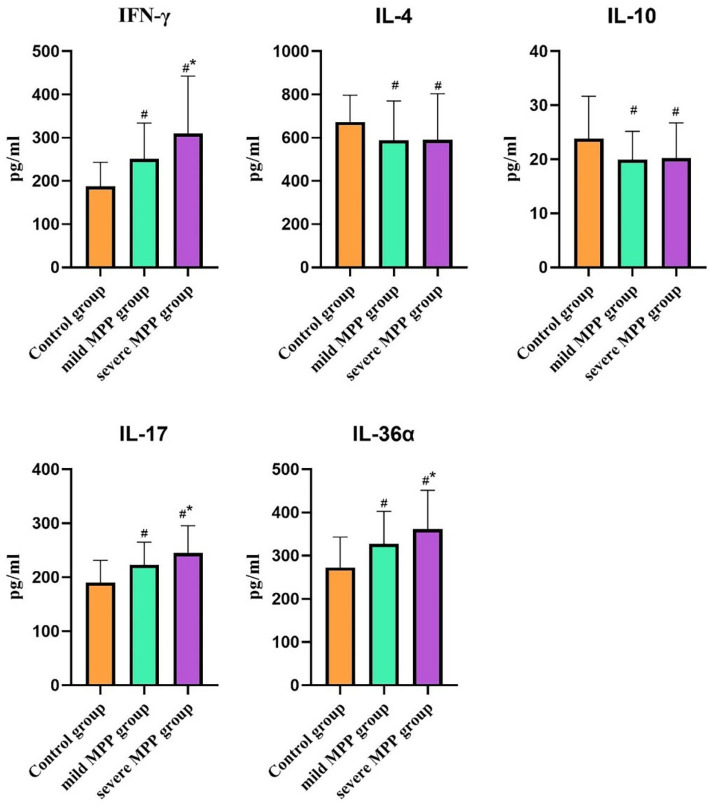
Correlation analysis between the Notch ligand DLL4 in the peripheral blood and clinical indicators in children with MPP. #*P* < 0.05 compared with the control group; *P < 0.05 compared with the mild MPP group.

The MPP group had significantly higher levels of plasma IFN-γ, IL-17, and IL-36α (all *P* < 0.001) and lower levels of IL-4 and IL-10 compared with the control group (*P* = 0.003 and 0.002). The severe MPP group had higher expression levels of plasma IFN-γ, IL-17, and IL-36α compared with the mild MPP group (*P* = 0.000, 0.011, and 0.000, respectively). However, no statistically significant differences in the expression levels of IL-4 and IL-10 were observed between the two groups (*P* = 0.955 and 0.810, respectively) (Figure 3).

### Correlation analysis between Notch ligand DLL4 in peripheral blood and clinical indicators in children with MPP

The expression levels of the Notch ligand DLL4 in the PBMCs of children with MPP were positively correlated with the hospitalization time and WBC count (r = 0.232 and 0.183, *P* = 0.008 and 0.039), but it had no significant correlation with the fever duration, neutrophil percentage, and CRP, IgG, IgA, and IgM levels (Table 1).

**Table 1:** Correlation analysis of the peripheral blood Notch ligand DLL4 and clinical indicators in the MPP group

Clinical indicators	Correlation coefficient (r)	DLL4
		*P*-value
Duration of fever (d)	0.133	0.134
Length of hospital stay (d)	0.232	0.008
WBC (1000/mL)	0.183	0.039
N (%)	0.062	0.485
CRP (mg/L)	−0.034	0.699
IgG (g/L)	0.118	0.185
IgA (g/L)	0.053	0.556
IgM (g/L)	−0.080	0.370

### Correlation analysis between Notch ligand DLL4 and lymphocyte subsets in the peripheral blood of children with MPP

**Figure 4:**
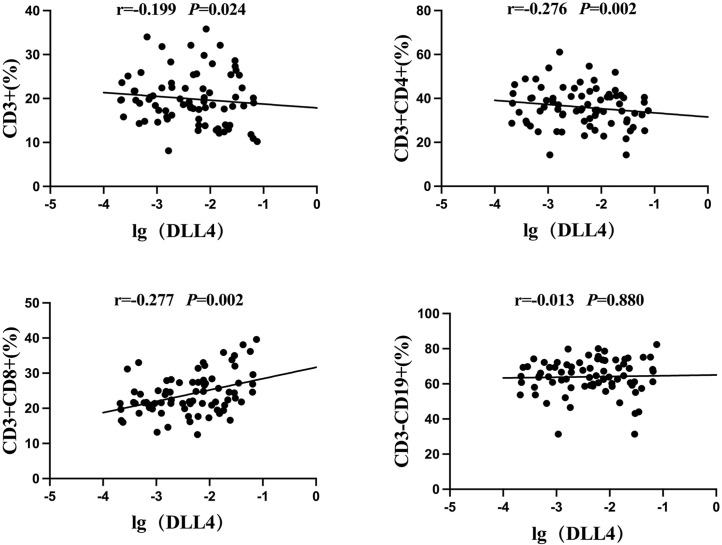
Correlation analysis of the Notch ligand DLL4 in the peripheral blood and lymphocyte subpopulations in children with MPP. r = correlation coefficient from the linear correlation analysis.

In the MPP group, the mRNA expression levels of the Notch ligand DLL4 in PBMCs were negatively correlated with the expressions of CD3+ and CD3+CD4+ cells (r = −0.199 and −0.276, *P* = 0.024 and 0.002), were positively correlated with CD3+CD8+ cells (r = 0.277, *P* = 0.002), and had no significant correlation with CD3-CD19+ cells (r = 0.013, *P* = 0.880) (Figure 4).

### Correlation analysis between Notch ligand DLL4 in peripheral blood and plasma IFN-γ, IL-4, IL-10, IL-17, and IL-36α levels in children with MPP

**Figure 5:**
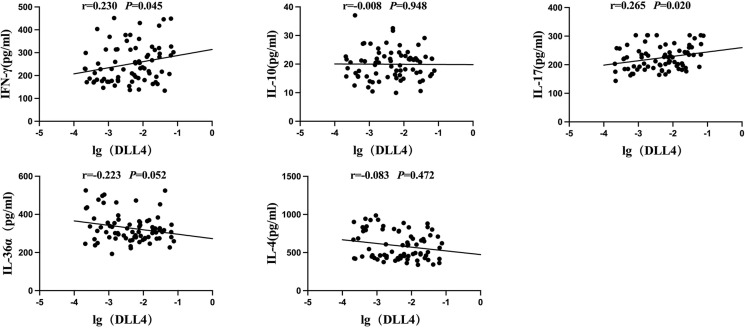
Correlation of the Notch ligand DLL4 in the peripheral blood with plasma IFN-γ, IL-4, IL-10, IL-17, and IL-36α levels. r = correlation coefficient from the linear correlation analysis.

In the MPP group, the mRNA expression levels of the Notch ligand DLL4 in PBMCs were positively correlated with plasma IFN-γ and IL-17 levels (r = 0.230 and 0.265, *P* = 0.045 and 0.020), and had no significant correlation with IL-4, IL-10, and IL-36α levels (r = −0.0830, −0.008, and −0.223; *P* = 0.472, 0.948, and 0.052) (Figure 5).

## DISCUSSION

This study shows that DLL4 is significantly upregulated in children with MPP, particularly in severe cases. Elevated DLL4 expression is positively correlated with plasma levels of IFN-γ, IL-17, and CD3+CD8+ T cells, and inversely correlated with CD3+ and CD3+CD4+ T cells. Furthermore, the DLL4 expression is associated with longer hospitalization and higher WBC counts. The findings suggest that DLL4 contributes to the immunopathogenesis of MPP by promoting Th1 and Th17 responses while suppressing Th2-mediated immunity.

Regarding clinical and demographic features, our findings indicated that mean age, fever duration, and hospitalization time were higher in children with severe MPP compared to those with mild MPP. Moreover, IgG and IgM concentrations were significantly elevated in children with severe MPP. Such findings suggest that severe MPP is more likely to occur in older children, possibly due to their more mature immune systems, which may elicit a stronger immune-inflammatory response following MP infection. When pathogens invade the body, white blood cells—known as the “white guards” of the human body—deform and penetrate the capillary walls, migrate to the infection site, and phagocytize the pathogens. Neutrophils, the primary effector cells of innate immunity, can also cross capillary walls, be recruited to infected tissues and organs, and phagocytize invading pathogens. CRP contributes to pathogen clearance by binding to ligands and activating the complement system and monocyte–macrophage pathways. Thus, peripheral blood WBC count, neutrophil percentage, and CRP levels reflect, to some extent, the degree of infection and inflammation in the body. In this study, the peripheral blood WBC count, neutrophil percentage, and CRP levels were significantly higher in children with severe MPP than in those with mild MPP, which were consistent with the findings of Fan *et al.* ^16^

Comparative analysis of clinical and immunological characteristics, supported by previous studies^9,11^, indicates that excessive immune-inflammatory responses — particularly involving both innate and adaptive immunity — play a key role in the pathogenesis of MPP, especially in severe cases. Neutrophils and macrophages mediate innate immunity, while dendritic cells (DCs) and Th cells are central to adaptive responses. The Notch signaling pathway, acting as a bridge between these systems, regulates the differentiation and development of immune cells, including T cells, B cells, and NK cells. We assessed Notch ligand expression in PBMCs, immune cell subsets, and cytokine levels (Th1, Th2, Th17) in children with MPP. DLL4 expression was significantly elevated in MPP patients, particularly in severe cases. These patients also showed decreased number of CD3^+^ and CD3^+^CD4^+^ T cells, and increased number of CD3^+^CD8^+^ and CD3^−^CD19^+^ cells. Moreover, levels of IFN-γ, IL-17, and IL-36α were elevated, whereas IL-4 and IL-10 were reduced, with more pronounced changes observed in severe cases. This indicates an immune imbalance favoring Th1/Th17 responses over Th2. DLL4 expression correlated positively with IFN-γ and IL-17 and negatively with CD3+ and CD3^+^CD4^+^ cells, suggesting that the Notch pathway, via DLL4, may drive immune dysregulation and inflammation in MPP.

The Notch signaling pathway influences the differentiation and development of immune cells, particularly T lymphocytes, and may participate in APC-mediated immune responses^17^. This pathway comprises ligands, receptors, CSL-DNA binding proteins and their effectors, and various regulatory molecules. The Notch signaling pathway in mammals includes five ligands (DLL1, DLL3, DLL4, Jagged1, and Jagged2) and four receptors (Notch1, Notch2, Notch3, and Notch4)^18-20^. Notch ligands are primarily expressed on APCs, among which DCs are important in T lymphocyte activation and the overall immune response. We analyzed the expression levels of Notch ligands in the peripheral blood of children in the MPP and control groups. DLL4 expression was significantly elevated in the MPP group, with a more pronounced increase in children with severe MPP, which suggests that the Notch signaling pathway may combine the Notch ligand DLL4 with Notch receptors, thereby influencing the pathogenesis of MPP in this population.

Adaptive immune cells primarily include Th cells and DCs. Th cells are differentiated from T lymphocytes, and the cell-mediated immune process by T lymphocytes can be used in identifying certain immunodeficiency and autoimmune diseases. Changes in the distribution and proportions of lymphocyte subsets can be accurate indicators of immune system dysfunction. Our findings showed that the levels of CD3^+^ and CD3^+^CD4^+^ cells in the peripheral blood of children with MPP were lower than those in the control group, with even greater reductions observed in children with severe MPP. In contrast, the proportions of CD3^+^CD8^+^ and CD3^–^CD19^+^ cells were higher in the MPP group compared to controls, and were more pronounced in the severe MPP group. As the major immune cells, lymphocytes are actively involved in the tissue damage associated with MP infection and are correlated with disease severity, consistent with the findings of Li *et al*.^10,21^.

IFN-γ is important in both innate and adaptive immunity and is mainly secreted by NK cells, as well as activated by CD4+T and CD8+T lymphocytes. IFN-γ shows multiple biological activities, including antiviral and antitumor effects, macrophage activation, cell differentiation, and immunomodulation. Its primary functions include regulating the expression of MHC-I and MHC-II molecules on immune cells, inducing the activation and proliferation of Th1 cells, and inhibiting of Th2 cell function^22^. Wang *et al*.^23^ reported that plasma IFN-γ levels are significantly elevated in children with MPP, suggesting that the immune response in these patients is predominantly Th1-mediated. Similarly, Yang *et al.* ^24^ proposed that IFN-γ contributes to the clearance of MP by activating macrophages and monocytes, and by enhancing the activity of NK cells and cytotoxic T lymphocytes, thereby indicating its involvement in both innate and adaptive immunity with a protective role during MP infection. Plasma IFN-γ levels were significantly higher in the MPP group compared to the control group, with the highest levels observed in children with severe MPP. Our findings suggest that IFN-γ, as a proinflammatory cytokine, contributes to the immune-mediated damage associated with MP infection and is closely related to disease severity.

IL-4, which is a Th2 cytokine primarily produced by activated T lymphocytes and monocytes, promotes T cell proliferation, supports B cell proliferation and differentiation, enhances their antigen-presenting capacity, and facilitates class switching from IgG to IgE. Shen *et al*.^25^ reported elevated plasma IL-4 levels during the acute stage of MPP in children compared to controls. In contrast, Qing *et al*.^26^ observed a reduction in IL-4 levels. Plasma IL-4 levels in the MPP group were substantially lower than those in the control group, with no significant difference between the severe and mild MPP groups; IL-4 enhances Th2-mediated immune responses while suppressing Th1 cell function^27^, which aids in antigen elimination and reduces inflammation. The observed reduction in IL-4 levels suggests a suppression of Th2-mediated immunity and a shift toward enhanced Th1 responses following Mycoplasma pneumoniae infection in children with MPP.

IL-10 is a cytokine with broad immunomodulatory effects, also known as cytokine synthesis inhibitory factor^28^, and is primarily secreted by Th2 cells but can also be produced by Th0 cells, B lymphocytes, mast cells, monocytes, macrophages, and keratinocytes. IL-10 is one of the major inhibitory inflammatory cytokines and serves as a key negative regulator of innate immunity; it inhibits the production and activity of proinflammatory cytokines, particularly those released by Th1 cells^29^. However, IL-10 plays a dual role in immune regulation: it limits tissue damage by suppressing excessive inflammation while also hindering immune responses by reducing the body’s ability to eliminate pathogens, potentially leading to immune suppression or “immune paralysis” in severe cases of MPP, thereby exacerbating infection^16^. Ding *et al*.^30^ found that children with MPP had decreased levels of plasma IL-10, especially in the acute phase and in severe cases, which suggests a weakened Th2 response following MP infection, leading to diminished negative regulation of Th1-mediated inflammation. In this study, plasma IL-10 levels were significantly lower in the MPP group than in the control group, with no significant difference observed between the severe and mild MPP subgroups. Zhang *et al*.^31^ reported elevated IL-10 levels during the acute phase of MPP, highlighting inconsistencies in current findings. The role of IL-10 in MP infection remains unclear, however, its involvement in the pathogenesis and progression of MPP is evident.

IL-17, primarily secreted by Th17 cells, is a recently identified cytokine belonging to the IL-12 family, its receptors are widely distributed throughout the body, and it exerts its effects by specifically binding to these receptors. The main biological functions of IL-17 include regulating the activity of T cells, B cells, and NK cells, promoting the release of various proinflammatory cytokines, and inducing chemokines. IL-17 plays a crucial role in enhancing autoimmune responses and promoting inflammation by these mechanisms^32^. Li *et al*.^33^ demonstrated elevated plasma IL-17 levels in a mouse model of MPP. Wang *et al*.^34^ also found significantly higher serum IL-17 levels in children with MPP compared to controls, reaching highest levels during the acute phase and correlating with the disease severity. Such findings suggest that IL-17 is involved in the pathogenesis of MPP. In this study, plasma IL-17 levels were significantly elevated in children with MPP, particularly in severe cases. These results are consistent with previous studies and indicate that IL-17 is closely associated with MP infection and that IL-17-mediated immune responses play a key role in the host defense. Children with MPP seem to show immune dysfunction, characterized by an imbalanced Th1/Th2 ratio and hyperactivation of Th17 cells. However, the mechanisms by which IL-17 contribute to MPP need to be further elucidated.

IL-36α is a newly identified proinflammatory cytokine that can be produced by various cell types, including T cells, monocytes, and keratinocytes. This is primarily distributed in tissues and organs such as the skin, lungs, and kidneys. Vigne *et al*.^35^ demonstrated that IL-36α can induce CD4^+^ T cells to secrete IFN-γ and IL-17, highlighting its importance in the immune response. In this study, plasma IL-36α levels were significantly higher in children with MPP compared to controls and were further elevated in the severe MPP group relative to the mild group. Such findings, along with previous studies, suggest that IL-36α contributes to the immune-inflammatory response following MP infection. Moreover, plasma levels of IFN-γ and IL-17 were also elevated in the MPP group compared to controls, which implies that IL-36α may exacerbate immune-mediated tissue damage by promoting the production of IFN-γ and IL-17.

The Notch signaling pathway is crucial in the differentiation of various immune cells^8^. Amsen *et al.* ^36^ found that the Notch ligands DLL1 and DLL4 can regulate and promote the differentiation of Th1 lymphocytes by binding to Notch receptors on the surface of T cells. This pathway may influence the differentiation and development of lymphocytes by the interaction between DLL1 or DLL4 ligands and their corresponding Notch receptors on target cells. We conducted a correlation analysis between Notch ligand levels in PBMCs from children with MPP and various clinical indicators to investigate whether the occurrence and progression of MPP are associated with the Notch signaling pathway. DLL4 expression was positively correlated with hospitalization duration and WBC count, but showed no significant correlation with fever duration, neutrophil percentage, or levels of CRP, IgG, IgA, and IgM. Additionally, no significant correlations were observed between clinical indicators and the expression levels of Jagged1, Jagged2, or DLL1. These findings suggest that the Notch signaling pathway — particularly DLL4 — may be important in the pathogenesis of MPP^36-39^.

A correlation analysis was also performed between Notch ligand levels and lymphocyte subsets. DLL4 showed the strongest correlation with lymphocyte subsets among the ligands and its expression was negatively correlated with the levels of CD3⁺ and CD3⁺CD4⁺ cells and positively correlated with the levels of CD3⁺CD8⁺ cells. However, it showed no significant correlation with the expression of the B lymphocyte surface marker (CD3⁻CD19⁺). These findings suggest that the Notch ligand DLL4 may be involved in the relative inhibition of CD3⁺ and CD3⁺CD4⁺ cells and the relative activation of CD3⁺CD8⁺ cells. According to the literature, the expression levels of CD3⁺, CD3⁺CD4⁺, and CD3⁺CD8⁺ cells are significantly associated with MPP progression^40^. Therefore, the Notch signaling pathway may influence the progression of MPP by modulating the function of these immune cells.

A correlation analysis was conducted between Notch ligand levels and plasma levels of IFN-γ, IL-4, IL-10, IL-17, and IL-36α. Among the ligands, DLL4 was positively correlated with the expression of IFN-γ and IL-17 but did not show significant correlation with IL-4, IL-10, or IL-36α. These findings suggest that the Notch signaling pathway may contribute to the pathogenesis of MPP by modulating T helper (Th) cell balance. Specifically, it may promote Th1-cell-mediated immune responses by enhancing IFN-γ production and regulating Th17 cells to increase IL-17 secretion. In turn, this can stimulate the release of various inflammatory mediators and induce chemokine production, leading to neutrophil recruitment and amplification of the inflammatory response.

The strength of this study lies in being the first to validate the association between the Notch ligand DLL4 and MPP infection. Regarding persistently increasing macrolide resistance in MPP ^13^, our findings provide novel therapeutic insights for MPP management. However, as one of the pioneering investigations examining the association between the Notch signaling pathway and MPP pathogenesis, our current research is constrained by a relatively small sample size and limited observation period. These factors may impede a comprehensive understanding of the dynamic regulatory patterns of this pathway during disease progression. Furthermore, the absence of well-characterized MPP animal models has restricted our ability to fully elucidate certain mechanistic aspects of this relationship.

## CONCLUSION

The Notch ligand DLL4 is highly expressed in children with MPP, with expression levels increasing in parallel with disease severity. DLL4 expression is significantly correlated with levels of CD3^+^, CD3^+^CD4^+^, CD3^+^CD8^+^ T cells, and with plasma concentrations of IFN-γ and IL-17. Our findings suggest that the Notch signaling pathway may be critical in the immune-inflammatory response following MP infection by modulating immune cell function and promoting the differentiation of Th1 and Th17 cells. However, this study has certain limitations that should be acknowledged.

## Data Availability

The complete anonymized dataset supporting the findings of this study is available at https://doi.org/10.48331/SCIELODATA.NKQAX2
