# Supplementary material for: Role of the Notch ligand DLL4 in the immune response of children with Mycoplasma pneumoniae pneumonia
Source: Rev Inst Med Trop Sao Paulo. 2025 Oct 3;67:e65. doi: 10.1590/S1678-9946202567065 (PMC12499519; doi:10.1590/S1678-9946202567065)
Supplement: Supplementary file 1 [file 1678-9946-rimtsp-67-S1678-9946202567065-suppl01.pdf]

REVISTA DO INSTITUTO DE MEDICINA TROPICAL DE SÃO PAULO

JOURNAL OF THE SÃO PAULO INSTITUTE OF TROPICAL MEDICINE

SUPPLEMENTARY MATERIAL

https://doi.org/10.48331/SCIELODATA.NKQAX2

Role of the Notch ligand DLL4 in the immune response of children with *Mycoplasma pneumoniae* pneumonia

Heting Dong[1]*, Zhiao Du[1]*, Yaru Liao[2]*, Jiying Sun[1], Huiming Sun[1], Peng Mo[1], Ge Dai[1], Li Huang[1], Feng Huang[1], Chuangli Hao[1], Zhengrong Chen[1], Yongdong Yan[1]

[1]Soochow University, Children's Hospital, Department of Respiratory Medicine, Suzhou, China

[2]Shandong University, Children's Hospital, Department of Respiratory Medicine, Jinan, China

*These authors contributed equally to the article

Correspondence to: Zhengrong Chen
Soochow University, Children's Hospital, Department of Respiratory Medicine, Suzhou, 215003, China
E-mail: chen_zheng_rong@outlook.com

Yongdong Yan
Soochow University, Children's Hospital, Department of Respiratory Medicine, Suzhou, 215003, China
E-mail: yyd_123@outlook.com

Received: 28 April 2025

Accepted: 18 July 2025

Supplementary Table S1 - Comparison of the clinical characteristics of children with MPP.

Group Clinical characteristics	Severe MPP group (n = 52)	Mild MPP group (n = 76)	Statistical value	*P* value
Age (yea)	5.50 ± 3.38	3.24 ± 2.66	t' = 4.039*	0.000
Sex (Male/Female)	25/27	47/27	χ^2 = 2.970	0.085
Duration of fever (d)	6.72 ± 5.89	2.58 ± 2.94	Z = −4.875	0.000
Length of hospital stay (d)	8.56 ± 2.87	7.34 ± 1.87	Z = −2.211	0.027
WBC (1000/mL)	11.25 ± 7.07	8.15 ± 3.30	Z = −2.729	0.006
N (%)	59.30 ± 19.31	48.83 ± 17.92	Z = −2.821	0.005
CRP (mg/L)	19.26(7.75~39.72)	6.61(2.40~15.64)	Z = −3.816	0.000
IgG (g/L)	6.53 ± 5.02	4.82 ± 3.80	Z = −2.043	0.041
IgA (g/L)	1.37 ± 1.19	3.58 ± 4.19	Z = −1.383	0.167
IgM (g/L)	1.35 ± 1.05	1.02 ± 0.54	Z = −2.060	0.039
Lobar pneumonia	41(78.8%)	17(22.4%)	χ^2 = 39.742	0.000
Bilateral lesions	10(19.2%)	15(19.7%)	χ^2 = 0.005	0.943
Pleural effusion	7(13.5%)	2(2.6%)	χ^2 = 5.540	0.019

*the *t*-test was used when the parameters were not homogeneous.

This is an open-access article distributed under the terms of the Creative Commons Attribution License.
